# *Copaifera mildbraedii* Desf.: Phytochemical Composition of Extracts, Essential Oil, and In Vitro Biological Activities of Bark

**DOI:** 10.3390/plants13060877

**Published:** 2024-03-19

**Authors:** Armel-Frederic Namkona, Rami Rahmani, Xavier Worowounga, Jean-Laurent Syssa-Magalé, Hubert Matondo, Jalloul Bouajila

**Affiliations:** 1 Faculté de Pharmacie de Toulouse, Université de Toulouse, Université Paul Sabatier, 118-Route de Narbonne, F-31062 Toulouse, France; namkof2000@yahoo.fr (A.-F.N.); worowoungax@yahoo.fr (X.W.); matondoster@gmail.com (H.M.); 2 Laboratoire d’Analyse, d’Architecture et de Réactivité des Substances Naturelles (LAARSN), Faculté des Sciences, Université de Bangui, Bangui BP 908, Central African Republic; syssamagalejl@hotmail.com; 3 Laboratoire de Recherche “Biodiversité, Molécules et Applications LR22ES02”, Institut Supérieur de Biologie Appliquée, Université de Gabes, Gabes 6072, Tunisia; rahmanirami2@gmail.com

**Keywords:** bioactivity, chemical composition, *Copaifera mildbraedii*, chromatography, principal components analysis

## Abstract

*Copaifera mildbraedii* Desf. is an evergreen tree with an umbrella-like crown. It is distributed from south-eastern Nigeria eastward to the Central African Republic (CAR). The aim of this study was to assess the chemical composition and biological activities of *C. mildbraedii* bark, as well as the chemical composition of the essential oil. Ethyl acetate (EtOAc) and methanol (MeOH) extracts showed a high total phenolic content (TPC) (149.9 and 148.8 mg GAE/g dry residue (dr), respectively), which was related to good antioxidant activity (DPPH) with an IC_50_ of 21.2 and 12.9 µg/mL, respectively. High-performance liquid chromatography coupled with diode array detector (HPLC-DAD) analysis revealed seven phenolic compounds with myricitrin (13.3 mg/g dr) and 2,4-dihydroxy-3,6-dimethyl benzoic acid (30.7 mg/g dr) as major compounds, while gas chromatography-mass spectrometry (GC-MS) analysis enabled detection of 13 volatile compounds (3 before and 10 after derivatization). Thirty compounds were identified in the essential oil, which corresponds to 65% of all identified compounds. Among the latter, E,E-farnesylacetone and γ-gurjunene were considered as major compounds (8.08 and 10.43%, respectively). The EtOAc extract showed a potent potential, simultaneously, against anti-acetylcholinesterase (AChE), anti-15-lipoxygenase (15-LOX), anti-xanthine oxidase (XOD), and cytotoxic (OVCAR) activities, whereas cyclohexane (CYHA) and dichloromethane (DCM) extracts showed a cytotoxic effect with high percentages of inhibition (95.2%).

## 1. Introduction

Tropical forests cover 7% of earth’s land surface [1]. They are the most biologically diverse environment in the world, and it is not surprising that they have been the source of many plant species, including medicinal plants [2]. However, compared to other vegetation types, tropical forests are poorly understood, mainly due to their structural and biological complexity and the extended time scale. This is largely due to a lack of long-term data on forest change and changes in determining factors. As a result, the Central African forests ecology remains hardly known and the literature on species diversity, tree growth, and phenological rhythms often lacks reference to African data [3]. Nevertheless, about 80% of the African population use the tree species as medicinal plants in order to treat against several diseases [4]. According to the World Health Organization (WHO), medicinal plants would be the best source to obtain a variety of drugs.

Natural substances are at the origin of the discovery of active ingredients in many fields: pharmacy, cosmetics, agri-food. The current trend favors their use over derived or synthetic chemicals. Another need is to analyze natural constituents and understand at the molecular level their role in ecosystems and on the well-being of living beings. The fields of environment, health and agriculture are largely concerned with the chemistry of natural substances. Phytochemistry provides a response to all these needs for these scientific fields to meet the demands of society.

*Copaifera* is a genus that belongs in the family Fabaceae that was first described by Marcgraf and Piso in 1638. The species that belong to this genus are native to the tropical regions of Latin America (mainly Argentina, Bolivia, and Brazil) and Western Africa (Congo, Cameroon, Guinea and Central African Republic) [5,6]. The *Copaifera* genus is commonly used in folk medicine in African countries. These plants display many pharmacological properties, including significant potential anti-inflammatory, analgesic and antimicrobial action [7].

*Copaifera mildbraedii* Desf. (*C. mildbraedii*) is a medicinal plant belonging to the Leguminosae family. In Africa, it is locally named Ovbia-Leke or Yama-Bilombi in the Central African Republic [6]. *Copaifera mildbraedii* is a large (up to 40 m) tree with an umbrella-like crown and cylindrical bole. It is also characterized by pinnate leaflets in 10 to 20 opposite pairs [8]. As a tree of the rainforest, it is found in Cameroon, Gabon, Democratic Republic of Congo, Nigeria and the Central African Republic [9]. In this latter, *C. mildbraedii* is used in folk medicine. In particular, the bark of this species is consumed for its anti-inflammatory and anti-tumor properties [10]. To the best of our knowledge several studies in the literature focus on the phytochemistry of oleoresin of the different *Copaifera* species, such as, *C. reticulate* [11], *C. multijuga*, *C. pubiflora* and *C. trapezifolia* [12]. However, despite the traditional medicinal virtues, little is known about the bark of *C. mildbraedii*, in terms of the extracts and essential oil chemical composition, as well as the different biological activities. The determination of the biochemical composition of *C. mildbraedii* bark has become of interest due to its consumption as a local beverage for many therapeutic reasons. The present study firstly focuses on the determination of the phytochemical composition of *C. mildbraedii* bark, collected from the tropical forest of Central African Republic, using spectrometric analysis (total phenolic content, total flavonoids content and condensed tannin content) and chromatographic analysis (HPLC and GC-MS). Secondly, an investigation of the biological activities of different extracts was conducted.

## 2. Results

### 2.1. Chemical Composition of Extracts

According to the literature, no studies have been reported before on the effect solvents on extraction yield, Total Phenolic Content (TPC), Total Flavonoid Content (TFC), Condensed Tannin Concentration (CTC), Total Anthocyanin Concentration (TAC) and Reducing Sugar Concentration (RSC) of *C. mildbraedii* bark.

#### 2.1.1. Yield Extraction, Total Phenolic Content (TPC), and the Reducing Sugar Content (RSC)

The yield and the TPC were determined for the different extracts of *C. mildbraedii*. The bark powder of *C. mildbraedii* was extracted using four organic solvents of increasing polarity (cyclohexane (CYHA), dichloromethane (DCM), ethyl acetate (EtOAc) and methanol (MeOH)). The apolar and the medium polar solvents (CYHA), (DCM) and (EtOAc) showed a very low yield extraction with percentages that did not exceed 1.0%. On the contrary, the polar solvent (MeOH) highlighted the highest extraction potent with a yield of 6.1% (Table 1). In general, the yields of polar extracts (MeOH) were about eight-fold higher than those of the non-polar extracts (CYHA and DCM). The present results were greater than that reported by Carmo et al. [13] in their work on the bark of *C. langsdorffii*. Moreover, among all the different extracts, only the MeOH extract exhibited a reducing sugar content of 269.2 milligrams of glucose equivalents per dry residue (mg GE/g dr) (Table 1). The other solvents were not able to extract the sugar compounds. The previous results of Singh and Madans [14] showed that the polar solvents were the best solvent for sugar compounds extracting, which confirms the results found in the current study. The TPC of *C. mildbraedii* extracts ranged from 10.0 to 149.0 milligrams of gallic acid equivalents per dry residue (mg GAE/g dr) (Table 1). Statistically, while there was a significative difference (*p* ≤ 0.05) between CYHA and DCM extracts, there was no significative difference (*p* > 0.05) between EtOAc and MeOH in terms of their TPC, compared to the other extracts. The EtOAc and MeOH extracts showed the highest TPC with 149.9 and 148.8 mg GAE/g dr, respectively (Table 1). The CYHA extract showed a low TPC of 10.0 mg GAE/g dr, followed by the DCM one with 23.5 mg GAE/g dr (Table 1). The TPC of the *C. mildbraedii* bark extracts was higher than found by Sharmin et al. [15], who reported a TPC of 60.8 mg GAE/g dr in MeOH extract of *A. chinensis* bark.

#### 2.1.2. Total Flavonoid, Anthocyanin and Condensed Tannin Contents (TFC, TAC, and CTC)

For the TFC, the EtOAc extract of *C. mildbraedii* showed the highest amount with 6.6 milligrams of quercetin equivalents per of dry residue (mg QE/g dr) (Table 2). The DCM and MeOH showed a close TFC (1.1 and 1.0 mg CE/g dr, respectively), with no significative difference (*p* ≤ 0.05), unlike CYHA extract, which showed no TFC. Statistically, there was a negative correlation between TPC and TFC (r = −13) (Table 3), which suggests that the majority of phenolic compounds in the different extracts were not flavonoids.

Regarding the CTC, among all tested extracts, the EtOAc extract was the only one which showed any significant content (Table 2). The CTC in this study, which was around 8 mg of catechin equivalents per dry residue mg CE/g dr, which was higher compared to the content found by Sujarnoko et al. [16]. They found a CTC in the *Acacia mangium* bark, which did not exceed 2 mg CE/g dr. Statistically, a good positive correlation was found between TPC and CTC (r = 0.58) (Table 3). These findings confirm that the condensed tannin were one of the main phenolic components [17,18]. For the TAC, *C. mildbraedii* extracts showed a modest content ranked from 0.3 to 1.9 mg of cyanidin-3-glucoside equivalents per g of dry residue (mg C3GE/g dr) (Table 2). Statistically, there was no significant (*p* > 0.05) difference between the two non-polar extracts (CYHA and DCM) in terms of their TAC, compared to the EtOAc and MeOH extracts (Table 2). The present results were higher than those found by Nitiema et al. [19] in their study on *Acacia gourmaensis* bark extracts.

### 2.2. Chromatographic Fingerprint Analyses using High-Performance Liquid Chromatography Coupled with Diode Array Detector (HPLC-DAD)

**Figure 1:**
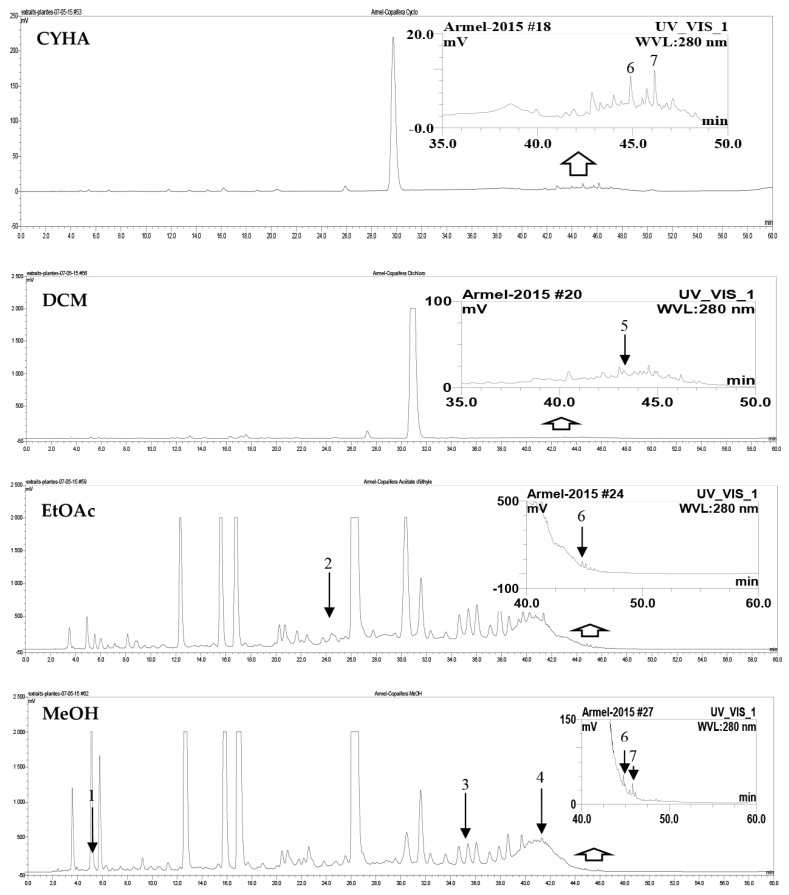
HPLC chromatograms of *C. mildbraedii* bark extracts. (CYHA: cyclohexane; DCM: dichloromethane; EtOAc: ethyl acetate; MeOH: methanol). Peaks: (1) 3-amino-4-hydroxy benzoic acid; (2) myricitrin; (3) 2,4-dihydroxy-3,6-dimethylbenzoic acid; (4) icariin; (5) 5,7-dihydroxy-4-propylcoumarin; (6) pinostilbene; (7) 4-hydroxy-3-propylbenzoic acid methyl ester.

**Figure 2:**
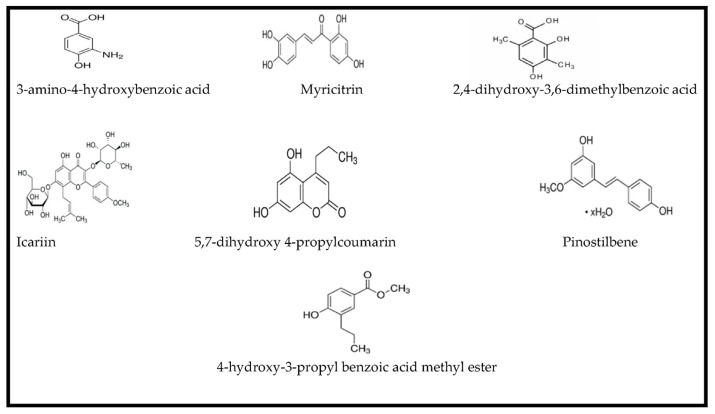
Chemical structures of the compounds detected in the extracts of *C. mildbraedii* bark extracts using HPLC-DAD analysis at 280 nm.

HPLC-DAD analysis of the different *C. mildbraedii* extracts was completed at 280 nm. The EtOAc and MeOH extracts chromatograms showed a similarity, in terms of profile and intensity (50 and 2500 mV) (Figure 1). These extracts displayed a large number of peaks with high intensity, which reach 2500 mV for both EtOAc and MeOH extracts. CYHA and DCM showed some similarity for the general profile with a very small number detected compounds. Regarding to the qualitative analysis, the different *C. mildbraedii* extracts has revealed seven different phenolic compounds in total (Figure 2; Table 4). While five phenolic compounds were identified in the MeOH extract, only one or two compounds were identified in the other extracts. The phenolic compounds 3-amino-4-hydroxybenzoic acid, 2,4-dihydroxy-3,6-dimethyl benzoic acid, icariin, pinostilbene and 4-hydroxy-3-propylbenzoic acid methyl ester were identified in the MeOH extract with the concentrations of 0.3, 30.7, 0.4, 0.5 and 0.2 mg/g dr, respectively (Table 4). This finding indicates that the MeOH extract was rich in polyphenols compounds. Interestingly, 2,4-dihydroxy-3,6-dimethyl benzoic acid showed the highest concentrations compared to the other identified compounds. For the CYHA and EtOAc extracts, two compounds of each extract were identified. Pinostilbene was the common compound between the two extracts with a small difference in concentration (1.2 mg/g dr for CYHA and 0.7 mg/g dr for DCM). In addition, myricitrin (13.3 mg/g dr) and 4-hydroxy-3-propylbenzoic acid methyl ester (0.5 mg/g dr) were also found in CYHA and EtOAc extracts, respectively (Table 4). However, 5,7-dihydroxy 4-propylcoumarin (0.5 mg/g dr) was the only identified compound in the DCM extract (Table 4).

### 2.3. Gas Chromatographic Analysis

#### 2.3.1. Chemical Composition of Essential Oil

According to the literature, no studies on the identification of *C. mildbraedii* bark essential oil components have been published. The components of the essential oil were identified by comparing their MS patterns and retention index (RI) with those of known chemicals published in the literature [27,28,29,30,31,32,33,34,35,36,37,38,39,40,41,42,43,44,45,46,47,48,49,50]. The chemical composition of the essential oil of *C. mildbraedii* bark was quantified using GC-FID and identified via GC-MS. Table 5 shows 30 compounds identified, representing 65% of all compounds detected in the GC-MS investigation of the essential oil, with 9 sesquiterpene hydrocarbons (26%), 7 oxygenated sesquiterpenes (22%) and 14 other compounds (17%). Among all the compounds identified, γ-gurjunene (10.43%), E,E-farnesylacetone (8.08%), and 8,14-cedranoxide (7.34%) were the predominant compounds, while Z-phytol (3.66%), γ-caryophyllene (3.61%), E-α-santalol (3.38%), α-acoradiene (2.79%), β-patchoulene (2.72%), Z-isolongifolanone (2.65%), widdrol (2.35%) and epiglobulol (2.00%) were the least predominant compounds. Most of sesquiterpenes presented here (Table 5), such as, δ-elemene, γ-caryophyllene, β-cedrene and α-guaiene, were described in previous research on the phytochemical profiles of species in the *Copaifera* genus [51]. However, more than one compounds were identified for the first time in the *Copaifera* bark essential oil, such as, α-longipinene, α-cubebene, β-patchoulene, Z-β-damascone, E-thujopsene and β-oplopenone.

#### 2.3.2. Volatile Compounds of Extracts

Gas chromatography coupled with mass spectrometry was used to identify the volatile compounds in the different extracts of *C. mildbraedii* bark. This is the first report which investigate the chemical composition of the *Copaifera* bark extracts. Only three volatile compounds (hydrocoumarin, 1,5-naphthyridin-4-ol and 1,3-di-O-acetyl-2,4,6 trimethylhexopyranose) were detected without derivation. Except 1,3-di-O-acetyl-2,4,6 trimethylhexopyranose, which was detected in the EtOAc extract, the other two compounds were detected in the MeOH extract (Table 6). Therefore, a silylation step was performed in order to identify more volatile compounds. No compounds (derivatized or not derivatized) were observed in DCM extract. On the other hand, this step led to the identification of 10 compounds in the other extracts (CYHA, EtOAc and MeOH) (Table 6). The volatile profile from the different extracts showed the presence of five organic compound classes: phenolic acid, naphthyridines, alcohols, sugar and fatty acid. Except glycerol and D-pinitol, which were detected in two extracts (EtOAc and MeOH), all the other compounds were detected only in one extract. GC-MS analysis showed a chemical composition difference between the different solvents, which depends on their polarities. Based on that, the MeOH extract was rich with alcohols and sugar compounds such as glycerol and glucopyranose (Table 6), whereas the fatty acid compounds (palmitic acid, oleic acid and stearic acid) were found in the CYHA extract.

### 2.4. Antioxidant Activity

**Figure 3:**
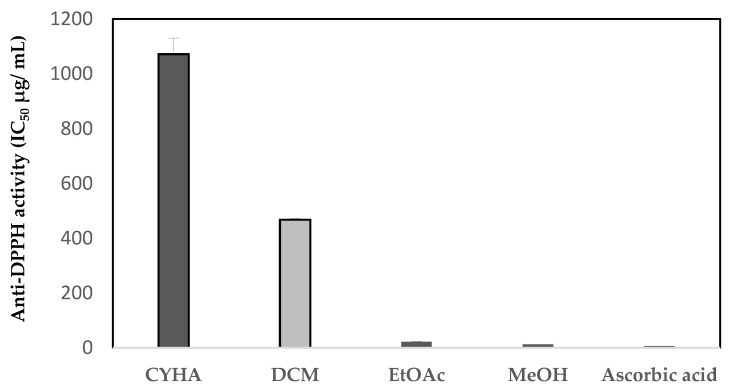
Antioxidant activity (IC_50_ µg/mL) of *C. mildbraedii* extracts using DPPH assay. (CYHA: cyclohexane; DCM: dichloromethane; EtOAc: ethyl acetate; MeOH: methanol).

Figure 3 showed the results of the antioxidant activity in the *C. mildbraedii* bark extracts, evaluated using the DPPH method. According to the previous knowledge, this is the first study about the antioxidant activity of extracts from species in the genus *Copaifera*. The results were expressed in IC_50_ value, which indicates that the highest anti-DPPH activity corresponded to the lowest concentration value. Statistically, there was no significant difference (*p* > 0.05) between MeOH, EtOAc and the ascorbic acid standard, compared to the CYHA and DCM extracts. The MeOH and EtOAc extracts showed a remarkable antioxidant activity with an IC_50_ of 12.9 and 21.5 (μg/mL), respectively (Figure 3). These concentrations were in the range of that obtained using the ascorbic acid, which was around 6.4 μg/mL. The other extracts showed a high IC_50_ value, which indicates their low anti-DPPH activity. The CYHA and DCM extracts showed an IC_50_ value of 1072.1 and 467.6 μg/mL, respectively (Figure 3). The present results were within the range of antioxidant values found by Pereira et al. [52] when working on *C. multijuga* bark extracts. They found that the polar extract (EtOH) exhibited an IC_50_ value of 22.9 μg/mL. Moreover, there was a positive high correlation between the TAC and their respective antioxidant activity (DPPH) (r = 0.86) (Table 3). Generally, a strong relationship was found between the phenolic compounds and antioxidant activity [53].

### 2.5. Anti-Acetylcholinesterase (AChE) Activity

The anti-AChE activity of *C. mildbraedii* bark has not been studied previously. There was no significant difference (*p* > 0.05) between EtOAc and MeOH extracts compared to the DCM extract. These extracts showed a good anti-AChE activity, with an inhibition percentage of 66.6 and 67.8%, respectively. However, the DCM and CYHA extracts showed low or no AChE inhibitory activity (Table 7). These data were in perfect correlation with the TPC with an r-value of 0.90 (Table 3). These findings suggested that the phenolic compounds present in *C. mildbraedii* were powerful compounds able to inhibit the AChE enzyme.

### 2.6. Anti-15-Lipoxygenaseoline (15-LOX) Activity

Organic extracts of *C. mildbraedii* bark were evaluated for their (15-LOX) enzyme inhibition and results are presented in Table 7. Statistically, the high TPC in the EtOAc extract was perfectly correlated with the 15-LOX enzyme inhibition (r = 0.99) (Table 3). These results indicated that these phenolic acids had stronger inhibitory activity against 15-LOX [54]. While the two non-polar extracts (CYHA and DCM) showed no AChE inhibitory activity, the other two extracts (EtOAc and MeOH) showed a low or a medium activity against 15-LOX. These extracts have a significant difference between them in terms of 15-LOX inhibition.

### 2.7. Anti-Xanthine Oxidase (XOD) Activity

The analysis was conducted with 50 μg/mL of each *C. mildbraedii* bark extract, and the results are shown in Table 7. Statistical analysis showed a significant difference (*p* ≤ 0.05) between the EtOAc and MeOH extracts in terms of their anti-XOD activity, compared to the other extracts (CYHA and DCM) (Table 7). The EtOAc extract exhibited the highest anti-XOD activity and its inhibition percentage was 53.0%, followed by the MeOH extract (40.9%). The CYHA and DCM extracts highlighted a low XOD activity, which does not exceed the 20%. Tung and Chang [55] tested the anti-XOD activity of Acacia confusa extracts. They found that the optimal inhibition of the XOD was registered with the EtOAc extract (59.0%) at 100 µg/mL, which was two times more concentrated than the EtOAc extract of *C. mildbraedii* used in the current study. No previous studies were conducted regarding the XOD inhibition by *Copaifera* species. None of the identified molecules were reported to exhibit an inhibition of the XOD activity in the literature.

### 2.8. Cytotoxic Activity

In order to investigate the cytotoxic activity of *C. mildbraedii* bark, the different extracts were evaluated against two ovarian cancer cell lines (IGROV and OVCAR) in vitro. Both of the cell lines were inhibited using the different *C. mildbraedii* bark extracts, with different percentages. *Copaifera mildbraedii* bark extracts showed a moderate to high cytotoxic inhibition effect ranked from 28.8 to 54.6% against OVCAR, and from 36.4 to 95.2% against IGROV (Table 8). Statistically, there was no significant difference between CYHA and DCM extracts, as well as between EtOAc and MeOH extracts in terms of IGROV cells inhibition. In addition, there was a significant difference between the different extracts, in terms of OVCAR cells line inhibition. Several previous studies showed the potent activity of coumarin (like 5,7-dihydroxy 4-propylcoumarin) and flavonoid (as icariin) compounds towards human ovarian cancer cells, such as OVCAR and IGROV [56]. Moreover, a positive correlation (r = 0.78) (Table 8) was found between CTC and the OVCAR cells inhibition on the one hand, and between TAC and IGROV (r = 0.84) (Table 3) on the other hand.

### 2.9. Principal Components Analysis (PCA)

**Figure 4:**
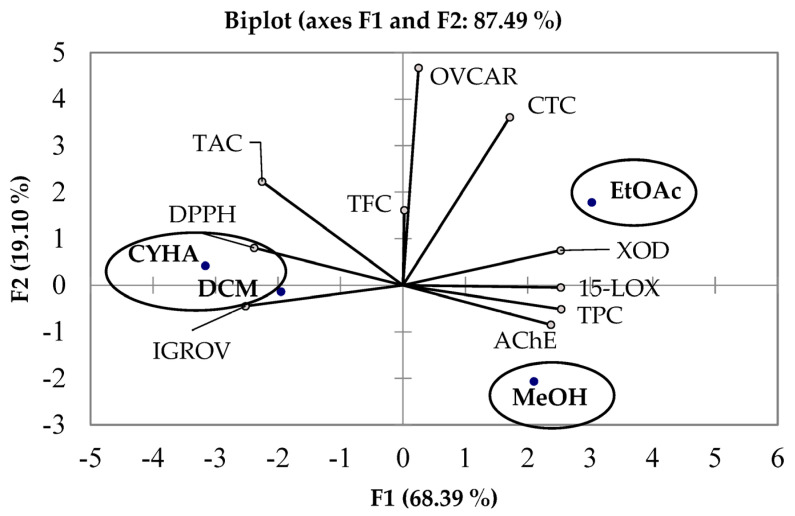
Principal components analysis biplot of biological activities *C. mildbraedii* extracts. CYHA: cyclohexane; DCM: dichloromethane; EtOAc; ethyl acetate; MeOH: methanol; TAC: total anthocyanin content; CTC: condensed tannin content; TFC: total flavonoid content; TPC: total phenolic content; DPPH: antioxidant activity; XOD; anti-xanthine activity; 15-LOX; anti-15-lipoxygenase; AChE: anti-acetylcholinesterase; (OVCAR, IGROV: cytotoxic activity.

Antioxidant and biological activities measurements of *C. mildbraedii* extracts were analyzed using PCA. The results of the PCA are shown in Figure 4. The two principal components (F1 and F2) explain 87.49% of the total data variance. From this analysis, the axes of inertia had been withheld, as seen in Table 9. The structuring of accessions showed 76.7% of the total variation (Figure 4). Axes were retained because they expressed 68.39% (F1) and 19.10% (F2). The loadings in the PCA loading plot express, at the same time, how well the principal components correlate with the original variables, and the correlations between the different activities and polyphenols (TPC, TFC and CTC). PC 1 correlated well, i.e., positively, with TPC, anti-15-LOX, anti-XOD activity and anti-AChE activity with a loading of 0.99, 0.99, 0.98 and 0.92, respectively (Table 10). However, PC 2 correlated well, with the cytotoxic activity (OVCAR) and CTC with the loading of 0.97, 0.75, respectively. However, it is less pronounced with TAC (r = 0.46) (Table 10). Overall, on the one hand, there was a positive correlation between TPC-15-LOX, TPC-AChE and TAC-XOD, having Pearson correlation coefficients values of 0.99, 0.90 and 0.97, respectively (Table 3). 

On the other hand, there was a correlation in the negative side of circle between TAC-DPPH, TAC-IGROV and DPPH-IGROV, having Pearson correlation coefficient values of 0.86, 0.84, 0.84 and 0.85, respectively (Table 3). Figure 4 showed the plots of the factor scores, and the oval forms grouped the different extracts in three classes. According to Figure 4, it seems that the CYHA extract possesses the highest antioxidant content (anti-DPPH), while the DCM extract possesses the highest potential against the IGROV cells line. Both activities were probably caused by the TAC. Moreover, EtOAc and MeOH extracts were located close to TPC, 15-LOX and XOD, giving an idea about the richness of this extract by phenolic compounds, which contributes to the inhibition of the two mentioned activities.

## 3. Materials and Methods

### 3.1. Chemicals

All chemicals used were of analytical reagent grade. All reagents were obtained from Sigma Aldrich (Saint-Quentin, France): ACTHi, acetylcholinesterase, catechin, CYHA, DCM, DMSO, DPPH, DTNB, EtOAc, Folin–Ciocalteu reagent (2N), gallic acid, HCl, KH_2_PO_4_, MeOH, MTT, NaOH, Na_2_HPO_4_, sodium carbonate, tamoxifen, XOD, and 15-LOX.

### 3.2. Plant Collection

The plant material, the bark of *Copaifera mildbraedii*, was collected from Boukoko, in the south of the Central African Republic (Central African Republic) in October 2011. A voucher specimen was deposited at the Laboratory of Analysis, Architecture and Reactivity of Natural Substances (Boukoko, Central African Republic) under the code AFM102011.

### 3.3. Plant Extraction (Extracts and Essential Oil)

The dried powder of *C. mildbraedii* bark was extracted using solvents of increasing polarity, with a constant solid/liquid ratio of 1:10 (*w*/*v*). The solid–liquid extraction was carried out for 4 h for each solvent, where the suspension was continuously mixed using a magnetic agitator. After a filtration step, the solvent was evaporated using a rotavapor under vacuum at 35 °C. The different residues obtained were evaluated for their phytochemical composition and their biological activities. 

For the essential oil, 1.2 kg of dry bark was used for extraction through hydrodistillation using a Clevenger-type apparatus during 4 h in the Laboratory of Analysis, Architecture and Reactivity of Natural Substances (Central African Republic). The essential oil was yellow (visually).

### 3.4. Total Phenolic Content (TPC)

The TPC of the different extracts of *C. mildbraedii* bark were quantified using the same method as Kohoude et al. [57], with slight modifications. In brief, 20 μL of extract was mixed with 100 μL of sodium carbonate (75 g/L in deionized water), and 100 μL of Folin–Ciocalteu reagent (0.2 N). The whole was stirred for 30 min and then incubated for 15 min. The absorbance was measured at 765 nm, using a microplate reader. The standard calibration curve was performed using gallic acid (0–115 µg/mL). Results were expressed in milligrams of gallic acid equivalents per gram of dry residue (GAE/g dr).

### 3.5. Total Flavonoid Content (TFC)

The TFC, in the various extracts, were estimated according to the Dowd method as described by Kohoude et al. [57]. In 96-well microplates, a volume of 100 μL of the diluted extract (0.5 mg/mL) was mixed with 100 μL 2% solution of aluminum trichloride (AlCl_3_) in MeOH. After an incubation of 15 min, the absorbance was measured at 415 nm against a blank sample (MeOH). Quercetin (2–10 µg/mL) was used as the reference compound to enable the drawing of the standard curve. The results were expressed in milligrams of quercetin equivalents per gram of dry residue (mg QE/g dr).

### 3.6. Determination of Condensed Tannins Content (CTC)

The CTC was determined through the vanillin method as described by Kohoude et al. [57], with minor modifications. The diluted solution (0.5 mg/mL) of each extract (50 μL) was mixed with 100 μL of vanillin solution (1% in 7 M H_2_SO_4_) in an ice bath. Then, this mixture was shaken and incubated at room temperature (20 to 25 °C) for 15 min. The absorbance of all samples was measured at 500 nm. Catechin was used as the reference to create the calibration curve and the results were expressed in milligrams of catechin equivalents per gram of dry residue (mg CE/g dr).

### 3.7. Determination of Total Anthocyanins Content (TAC)

The TAC contained in the various extracts of *C. mildbraedii* was determined using the pH differential absorbance method as described by Kohoude et al. [57]. Two buffer solutions were prepared: The first solution consisted of hydrochloric acid and potassium chloride (pH 1.0 and 0.2 M, respectively). The second buffer solution was a mixture of acetic acid and sodium acetate (pH 4.5 and 1 M, respectively). Briefly, 180 μL of the buffer solution was added to 20 μL of extract. The reading was made on two wavelengths at 510 and 700 nm after 15 min of incubation. The following equation was applied for the calculation:

TAC = [(A510 − A700) pH 1.0 − (A510 − A700) pH 4.5].

The results were expressed in milligrams of cyanidin-3-glucoside equivalents per gram dry residue (mg C3GE/g dr).

### 3.8. Determination of Reducing Sugars Content (RSC)

The RSC quantification of *C. mildbraedii* extract was carried out according to the procedure used by Kohoude et al. [57], with minor modifications. The reaction mixture contained 100 μL of each extract (0.5 mg/mL) and 150 μL of DNS solution (0.05 M). After shaking and incubation for 5 min in a water bath at 100 °C, 750 μL of water was added. The absorbance of the mixture was measured, after a second stirring, at 530 nm against a blank. The reducing sugar amount was determined in milligrams of glucose equivalent per gram of dry residue (mg GE/g dr).

### 3.9. Chromatographic Fingerprint Analyses using High-Performance Liquid Chromatography Coupled with Diode Array Detector (HPLC-DAD)

HPLC analysis was performed using Ultimate 3000 Pump-Dionex and Thermos Separation model UV-150 detectors (Thermo Fisher Scientific, Waltham, MA, USA) as reported by Rahmani et al. [58]. The separation was completed on a column of RP-C18-type (25 cm × 4.6 mm, 5 μm) at room temperature (20 to 25 °C). Elution was performed at a flow rate of 1.2 mL/min, using a mobile phase that consisted of acidified water (pH 2.65) (solvent A), and acidified water/acetonitrile (ACN) (20:80 *v*/*v*) (solvent B). The samples were eluted according to the following linear gradient: from 0.1 B to 30% B for 35 min, from 30 B to 50% B for 5 min, from 50 B to 99.9% B for 5 min, and finally a return to 0.1% B for 15 min. All the extracts were prepared at the concentration of 20 mg/mL in the mixture acidified water/ACN (80:20 *v*/*v*), then filtered through a filter (Sigma Aldrich, Millex-HA 0.45 μm filter, St. Quentin, France). After that, 20 μL of each extract was injected and the detection was made at a wavelength of 280 nm. The phenolic compounds were identified through a comparison of the retention time of known standards and then quantified using their calibration curves.

### 3.10. Gas Chromatographic Analysis

#### 3.10.1. Essential Oil Analysis

The chemical identification and quantification of the essential oil were completed according to the previous work of Kohoude et al. [57]. Gas chromatography-flame ionization detection (GC-FID) analyses was carried on a Varian Star 3400C × chromatograph (Les Ulis, France) fitted with a fused silica capillary DB-5MS column (5% phenylmethylpolysyloxane, 30 × 0.25 mm, film thickness 0.25 µm). Chromatographic conditions initially were began from 60 to 260 °C, then the temperature rose with a gradient of 5 °C/min and 15 min isotherm at 260 °C. After that, a second gradient was applied to 340 °C at 40 °C/min. For analysis reasons, petroleum ether was used to dissolve the essential oil. One microliter was injected in the split mode ratio of 1:10 and the helium was used as the carrier gas at 1 mL/min. The injector was operated at 200 °C. For the gas chromatography-mass spectrometry (GC-MS) system (Varian Saturn 2000 ion trap GC/MS with CP-3800 GC), it was used with the same chromatographic conditions as GC-FID. The MS system was adjusted for an emission current of 10 µA and electron multiplier voltage between 1400 and 1500 V. The trap temperature was 250 °C and that of the transfer line was 270 °C and the mass scanning was from 40 to 650 amu. The identification of the compounds was performed through (i) comparison of their retention index (RI) relative to C_5_-C_24_ n-alkanes obtained on a nonpolar DB-5MS column, with those provided in the literature and (ii) by comparison of their mass spectra with those recorded in NIST 08, reported in published articles or using co-injection of available reference compounds. The percentage composition of the essential oil was measured using the normalization method from the GC peak areas, assuming identical mass response factor for all compounds.

#### 3.10.2. Volatile Compounds of Extracts

The volatile compounds identification from the different organic extracts, before or after derivatization, was carried with the same equipment GC-MS. The analysis was carried out following this gradient: 5 min at 60 °C, then 60–270 °C at 15 °C/min, 6 min at 270 °C, from 270 to 300 °C at 50 °C/min and finally stable at 300 °C for 4.5 min. The entire chromatographic program lasted 30 min. The derivatization method consisted of the method as described by Kohoude et al. [57], with minor modifications.

### 3.11. Antioxidant Activity

The anti-radical activity of the extracts was determined through the method as described by Kohoude et al. [57], slightly modified. In a 96-well microplate, 20 μL of each extract was added to 180 μL of the methanolic DPPH solution (0.2 mM). The mixture was stirred for 30 s and then incubated for 30 min in the dark. The reading was made at 524 nm. The inhibition percentage of the extracts was calculated using the following equation:

% inhibition = 100 × (Ablank − Asample)/Ablank

The antioxidant activity of the extract was expressed as IC_50_, which defines the concentration of the extract that reduces the free radical by 50% (DPPH). Ascorbic acid was used as a standard.

### 3.12. Anti-Acetylcholinesterase (AChE) Activity

The AChE activity was determined using the Ellman colorimetric method as previously described by Kohoude et al. [57], with some modifications. In a 96-well microplate, 50 μL of 0.1 mM sodium phosphate buffer (pH = 7.5), 125 μL of DTNB, 25 μL of diluted plant extract (0.5 mg/mL) and 25 μL of enzyme solution were mixed and incubated for 15 min at 25 °C. Thereafter, 25 μL of ACTHi was added. Then, the final blend was incubated for 25 min at room temperature, and then the absorbance was measured at 421 nm. Galantamine has been used as a reference. The Ablank was measured without extract. The enzyme activity inhibition percentage was calculated as: % inhibition = 100 × (Ablank − Asample)/Ablank.

### 3.13. Anti-15-Lipoxygenaseoline (15-LOX) Activity

Linoleic acid (substrate) was oxidized in vitro to a conjugate diene using 15-lipoxygenase. The anti-15-LOX activity was evaluated via the spectrophotometric measurement of the conjugated diene at 234 nm [57]. The different diluted extracts (20 µL) were mixed with 170 μL of sodium phosphate buffer (pH = 7.4), 60 µL of linoleic acid (3.5 mM) and 20 μL of enzyme solution (15-LOX). The mixture was incubated at 25 °C for 10 min and the absorbance was determined at 234 nm. The percentage of the enzyme activity was plotted against the concentration of each extract. The nordihydroguaiaretic acid (NDGA) was used as a reference. The Ablank was measured without extract. The enzyme activity inhibition percentage was calculated as: % inhibition = 100 × (Ablank − Asample)/Ablank.

### 3.14. Anti-Xanthine Oxidase (XOD) Activity

The XOD activity was measured spectrophotometrically using the procedure of Kohoude et al. [57], slightly modified. The xanthine solution (1 mM) was prepared by dissolving this substrate in 25 mL of 0.1 mM sodium phosphate buffer (pH = 7.5). The xanthine oxidase enzymatic solution was prepared by diluting xanthine oxidase enzyme (1 U/mL) to a final concentration of 0.1 U/mL. Briefly, 50 μL of diluted plant extract (0.2 mg/mL), 60 μL of 70 mM sodium phosphate buffer (pH = 7.5) and 30 μL of the enzymatic solution were mixed together, giving a final extract concentration of 50 mg/L in each well of a 96-well microplate. After 25 min of incubation, 60 μL of substrate solution was added and then the absorbance was measured at 295 nm after 5 min. Allopurinol was used as a reference. The Ablank was measured without extract. The XOD activity was expressed as the inhibition percentage of XOD enzyme, calculated as: % inhibition = 100 × (Ablank − Asample)/Ablank.

### 3.15. Cytotoxic Activity

The anti-proliferation activity of the different extracts of *C. mildbraedii* bark was estimated against two human ovarian cancer cell lines: IGROV and OVCAR (American-Type Culture Collection) as described by Kohoude et al. [57]. Cells were distributed in 96-well plates at 3 × 10^4^ cells/well in 100 μL. After that, 100 μL of the corresponding culture medium (Dulbecco’s Modified Eagle Medium (DMEM)) containing sample at various concentrations were added. Cell growth was estimated using the MTT assay. MTT is a water-soluble tetrazolium salt with a yellow coloration. Metabolically active cells are able to convert the dye to water-insoluble dark blue formazan through reductive cleavage of the tetrazolium ring. The extracts were re-solubilized in the dimethyl sulfoxide (DMSO) followed by dilution in the buffer, whereby the DMSO does not exceed 1%. Doxorubicin was used as a positive control. The cells activity inhibition percentage was calculated as: % inhibition = 100 × (Ablank − Asample)/Ablank.

### 3.16. Statistical Analysis

In this study, the measurements were made in three repetitions. Analysis of variance (one-way ANOVA) using SPSS 20.1 (version 20.0.2004) was able to calculate the data for significance. Tukey’s test was used to determine statistical differences between solvents. The determination of the relationship between TPC, TFT, TAC, CTC and biological activities was assessed using Pearson correlation analysis (r). Principal component analysis (PCA) was performed using XLSTAT (version 2014.5.03) for the visualization of discrimination between different parameters.

## 4. Conclusions

To the best of our knowledge, this study is the first work to highlight biological activities of *C. mildbraedii* bark extracts as well as their chemical composition and essential oil. The physico-chemical composition of *C. mildbraedii* bark showed that EtOAc extract highlighted the highest amount of TPC, TFC, CTC and TAC. In addition, *C. mildbraedii* bark showed a moderate anti-15-LOX activity and an active AChE and cytotoxic effect. GC-MS/GC-FID analysis enabled us to identify new compounds, such as α-longipinene and α-cubebene in the essential oil of *C. mildbraedii* bark. Among the identified phenolic compounds, 2,4-dihydroxy-3,6-dimethyl benzoic acid showed the highest concentration, which exceeded the level of 30 mg/g dr. Overall, PCA proved that there was a positive correlation between TPC and 15-LOX on the one hand, and between TPC and AChE on the other hand. These data suggest that this plant could be a valuable source of secondary metabolites that have beneficial properties and a promising source of health products.

## Figures and Tables

**Table 1:** Extraction yield and chemical composition of *Copaifera mildbraedii* extracts.

Extracts	Yields (%)	TPC mg GAE/g dr	RSC mg GE/g dr
Cyclohexane	0.7	10.0 ± 0.4 ^c^	nd
Dichloromethane	0.5	23.5 ± 0.5 ^b^	nd
Ethyl acetate	0.7	149.9 ± 6.0 ^a^	nd
Methanol	6.1	148.8 ± 1.9 ^a^	269.2 ± 2.6

nd: not detected. ^a, b, c^: the different superscripts in the same column represent significant differences between the TPC values according to Tukey’s test when comparing the extracts of the same species (*p* ≤ 0.05).

**Table 2:** Total flavonoids, anthocyanin, and condensed tannin contents.

Extracts	TFC mg QE/g dr	CTC mg CE/g dr	TAC mg (C3GE)/g dr
Cyclohexane	nd	nd	1.9 ± 0.1 ^a^
Dichloromethane	1.1 ± 0.1 ^b^	nd	1.7 ± 0.1 ^a^
Ethyl acetate	6.6 ± 0.3 ^a^	8.4 ± 0.2	0.9 ± 0.0 ^b^
Methanol	1.0 ± 0.8 ^b^	nd	0.3 ± 0.0 ^c^

nd: not detected. ^a, b, c^: the different superscripts in the same column represent significant differences between the TFC and CTC, and TAC values according to Tukey’s test when comparing the extracts of the same species (*p* ≤ 0.05).

**Table 3:** Correlation matrix (Pearson (n)).

Variables	TPC	CTC	TFC	TAC	DPPH	AChE	15-LOX	IGROV	OVCAR	XOD
TPC	1	0.58	−0.13	−0.93	0.90	0.90	0.99	−0.98	0.02	0.97
CTC	0.58	1	0.25	−0.25	0.50	0.48	0.65	−0.73	0.78	0.77
TFC	−0.13	0.25	1	0.25	0.26	0.27	−0.15	0.10	0.09	−0.02
TAC	−0.93	−0.24	0.25	1	0.86	−0.86	−0.89	0.84	0.33	−0.81
DPPH	−0.90	−0.50	−0.26	0.86	1	−1.00	−0.86	0.85	0.15	−0.86
AChE	0.90	0.48	0.27	−0.86	1.00	1	0.86	−0.84	−0.16	0.86
15-LOX	0.99	0.65	−0.15	−0.89	0.86	0.86	1	−0.99	0.13	0.98
IGROV	−0.98	−0.73	0.10	0.84	−0.85	−0.84	−0.99	1	−0.22	0.99
OVCAR	0.02	0.78	0.09	0.33	−0.15	−0.16	0.13	−0.22	1	0.27
XOD	0.97	0.77	−0.02	−0.81	−0.86	0.86	0.98	0.99	0.27	1

**Table 4:** Quantification of the compounds detected in the extracts of *C. mildbraedii* bark extracts using HPLC-DAD analysis at 280 nm.

N°	Rt (min)	Compounds	Concentration (mg/g dr)				References
			CYHA	DCM	EtOAc	MeOH	
**1**	2.2	3-amino-4-hydroxybenzoic acid	nd	nd	nd	0.3	[20]
**2**	25.3	Myricitrin	nd	nd	13.3	nd	[21]
**3**	35.2	2,4-dihydroxy-3,6-dimethyl benzoic acid	nd	nd	nd	30.7	[22]
**4**	42.0	Icariin	nd	nd	nd	0.4	[23]
**5**	43.4	5,7-dihydroxy 4-propylcoumarin	nd	1.5	nd	nd	[24]
**6**	44.7	Pinostilbene	1.2	nd	0.7	0.5	[25]
**7**	46.13	4-hydroxy-3-propyl benzoic acid methyl ester	0.5	nd	nd	0.2	[26]

nd: not detected.

**Table 5:** Chemical composition of the essential oil of barks of *C. mildbraedii*.

N°	Compounds	Area (%)	Chemical Formula	RI	RI from the Litterature
**1**	δ-elemene	0.29	C_15_H_24_	1339	1329 [27]
**2**	α-longipinene	0.33	C_15_H_24_	1351	1351 [28]
**3**	α-cubebene	0.44	C_15_H_24_	1360	1361 [29]
**4**	Cyclosativene	0.54	C_15_H_24_	1371	1371 [30]
**5**	β-patchoulene	2.72	C_15_H_24_	1377	1386 [31]
**6**	Z-β-damascone	0.17	C_13_H_20_O	1383	1386 [32]
**7**	Isolongifolene	0.40	C_15_H_24_	1388	1388 [33]
**8**	γ-caryophyllene	3.61	C_15_H_24_	1406	1406 [34]
**9**	Isocaryophyllene	0.65	C_15_H_24_	1413	1417 [35]
**10**	β-cedrene	1.00	C_15_H_24_	1418	1418 [31]
**11**	E-thujopsene	1.23	C_10_H_14_O_2_	1428	1428 [36]
**12**	α-guaiene	1.14	C_10_H_16_	1439	1444 [35]
**13**	E-4,5-muroladiene	0.90	C_10_H_14_O_2_	1451	-
**14**	α-acoradiene	2.79	C_15_H_24_	1465	1466 [37]
**15**	γ-gurjunene	10.43	C_21_H_32_O_4_	1472	1473 [37]
**16**	2,6-dibutyl-4me-phenol	1.73	C_15_H_24_O	1502	1508 [38]
**17**	8,14-cedranoxide	7.43	C_15_H_24_O	1537	1542 [39]
**18**	Tridecan-1-ol	0.95	C_13_H_28_O	1576	1578 [40]
**19**	β-oplopenone	1.53	C_15_H_24_O	1584	1606 [39]
**20**	Epiglobulol	2.00	C_15_H_26_O	1587	1582 [40]
**21**	Widdrol	2.35	C_15_H_26_O	1597	1584 [41]
**22**	Cedrenol	1.14	C_15_H_24_O	1603	1606 [42]
**23**	Z-isolongifolanone	2.65	C_15_H_24_O	1620	1612 [43]
**24**	10-methylundecan-4-olide	0.65	C_12_H_25_O_2_	1657	1659 [44]
**25**	E-α-santalol	3.38	C_15_H_24_O	1677	1679 [45]
**26**	Blumenol C	0.69	C_13_H_20_O_3_	1704	1713 [46]
**27**	Methyl palmitate	0.97	C_17_H_34_O_2_	1911	1911 [47]
**28**	Isophytol	1.35	C_20_H_40_O	1922	1938 [48]
**29**	E,E-farnesylacetone	8.08	C_18_H_28_O	1932	1921 [49]
**30**	Z-phytol	3.66	C_20_H_40_O	2113	2113 [50]
Total identified (%)			65		
Sesquiterpene hydrocarbons (%)			26		
Sesquiterpene oxygenated (%)			22		
Others (%)			17		

**Table 6:** Volatile compounds identified using GC-MS before and after derivatization of *C. mildbraedii* extracts.

N°	Rt (min)	Compounds	CYHA	DCM	EtOAc	MeOH
Before derivatization						
**1**	13.01	Hydrocoumarin	nd	nd	nd	++
**2**	13.72	1,5-naphthyridin-4-ol	nd	nd	nd	++
**3**	14.56	1,3-di-O-acetyl-2,4,6 trimethylhexopyranose	nd	nd	+++	nd
After derivatization						
**1**	11.50	Glycerol	nd	nd	+++	+++
**2**	15.29	Erythrose oxime	nd	nd	nd	+++
**3**	15.93	D-erythro-Pentonic acid, 3-deoxy-2-C-(hydroxymethyl)-	nd	nd	nd	+
**4**	15.98	D-psicofuranose	nd	nd	nd	+
**5**	16.18	D-pinitol	nd	nd	+++	+++
**6**	17.12	Glucopyranose	nd	nd	nd	+++
**7**	17.53	Palmitic acid	++++	nd	nd	nd
**8**	18.59	Oleic acid	++	nd	nd	nd
**9**	18.74	Stearic acid	++	nd	nd	nd
**10**	19.46	Cis-5,8,11-eicosatrienoic acid	+++++	nd	nd	nd

Rt: retention time; nd: not detected; +++++: exrtemely abundant; ++++: high abundant; +++: very abundant; ++: moderately abundant; +: weakly abundant.

**Table 7:** Anti-AChE, anti-15-LOX and anti-XOD activities of *C. mildbraedii* extracts.

Extract	Anti-AChE Activity (%)	Anti-15-LOX Activity (%)	Anti-XOD Activity (%)
CYHA	na	na	14.5 ± 1.6 ^c^
DCM	39.5 ± 0.7 ^b^	na	17.1 ± 1.2 ^c^
EtOAc	66.6 ± 0.5 ^a^	39.7 ± 5.0 ^a^	53.0 ± 3.5 ^a^
MeOH	67.8 ± 4.2 ^a^	25.9 ± 3.3 ^b^	40.9 ± 0.7 ^b^
Galantamine	95.9 ± 0.2		
NDGA		95.1 ± 1.8	
Allopirinol			92.5 ± 1.9

na: not active; the different superscript in the same column means significant difference (*p* ≤ 0.05).

**Table 8:** Cytotoxic activity of *C. mildbraedii* extracts against OVCAR and IGROV cell lines.

Extracts	OVCAR (%)	IGROV (%)
CYHA	45.9 ± 2.8 ^b^	95.2 ± 4.3 ^a^
DCM	35.9 ± 4.5 ^c^	95.2 ± 4.3 ^a^
EtOAc	54.6 ± 1.3 ^a^	36.4 ± 3.9 ^c^
MeOH	28.8 ± 3.5 ^c^	50.7 ± 1.0 ^b^
Tamoxifen	77.4 ± 7.6 ^a^	57.8 ± 1.8 ^b^

The different superscript in the same column means significant difference (*p* ≤ 0.05).

**Table 9:** Contribution of the variables (%).

	F1	F2
TPC	14.29	0.59
CTC	6.51	29.06
TFC	0.001	5.81
TAC	11.35	11.01
DPPH	12.67	1.44
AChE	12.49	1.61
15-LOX	14.21	0.005
IGROV	14.17	0.45
OVCAR	0.14	48.71
XOD	14.17	1.25

**Table 10:** Correlations between variables and factors.

	F1	F2
TPC	0.99	−0.11
CTC	0.67	0.75
TFC	0.01	0.33
TAC	−0.88	0.46
DPPH	−0.93	0.17
AChE	0.92	−0.18
15-LOX	0.99	−0.01
IGROV	−0.98	−0.09
OVCAR	0.10	0.97
XOD	0.99	0.15

## Data Availability

Data are contained within the article.
